# Liver regeneration governed by zonated PIEZO1 mechanotransduction

**DOI:** 10.1186/s13619-026-00305-w

**Published:** 2026-10-10

**Authors:** Lin Xie, Chen Zhang, Yonglong Wei, Wenxiu Ning

**Affiliations:** Yunnan Key Laboratory of Cell Metabolism and Diseases, Center for Life Sciences, School of Life Sciences, Yunnan University, Kunming, China

## Abstract

Mechanical forces are recognized as regulators of organ development, homeostasis, and regeneration, yet how physical cues are sensed in a region-specific manner within the liver remains unclear. In a *Science* article, Zhang and colleagues identify DPP4-positive zone 2 hepatocytes as a mechanosensory population, demonstrating that mechanical stress triggers the regenerative response through PIEZO1-mediated mechanotransduction. PIEZO1 activation in these cells induces NFATC3-dependent IGFBP2 expression, establishing a molecular link between tissue mechanics and spatially coordinated proliferation. This work provides a mechanistic explanation for why regeneration originates from the mid-lobule and introduces a druggable mechanotransduction axis that might be enhanced to accelerate liver repair.

## Main text

Mechanical forces, including tissue tension, shear stress, and matrix stiffness, are recognized as regulators of organ development, homeostasis, and regeneration. Cells detect them through complementary mechanosensing systems that respond to distinct inputs. Integrins mediate cell-matrix adhesion and couple the matrix to the actin cytoskeleton, whereas E-cadherin-based adherens junctions mediate cell-cell adhesion and couple neighboring actomyosin networks. PIEZO1, in contrast, is a mechanically activated ion channel that converts membrane tension into calcium influx. These signals are further transmitted to the nucleus through the LINC complex, while YAP and TAZ, as effectors, respond to mechanical cues and regulate gene expression. Conversely, forces can also be generated through the inside-out RhoA-ROCK1-actomyosin contractility axis, which modulates cellular tension and cortical stiffness. These outside-in and inside-out pathways converge to translate physical cues into biochemical outputs that influence proliferation, differentiation, and apoptosis (Di et al. 2023).

Evidence highlights the roles of mechanical force signaling across tissues. In skin, increasing suprabasal actomyosin contractility enhances basal cell proliferation (Ning et al. 2021), whereas PIEZO1 loss induces hair follicle stem cells to exit quiescence and enter anagen (Wang et al. 2025). In the colon, PIEZO1 restricts proliferation via SCD1/Wnt, and its inhibition promotes epithelial renewal (Fang et al. 2025). Likewise, the liver is subject to mechanical regulation and possesses remarkable regenerative capacity. For example, partial hepatectomy increases portal flow, shear stress, and tissue tension in the remnant liver, and these mechanical changes are required to drive regeneration (Marubashi et al. 2004). These changes perturb sinusoidal mechanical homeostasis. Liver sinusoidal endothelial cells (LSECs) can sense the shear stress through GPCR-dependent Ca^2^⁺ influx, downregulate TGFβ1, and thereby permit hepatocyte proliferation (Ishikawa et al. 2021). In addition, LSECs function as a spatiotemporal rheostat, as Ang2 downregulation releases a TGFβ1 brake on hepatocytes and its later recovery promotes VEGFR2-dependent angiogenesis (Hu et al. 2014). Integrins also contribute to this mechanotransduction. Loss of hepatocyte β1-integrin impairs EGFR and c-Met signaling and compromises regeneration (Speicher et al. 2014), whereas endothelial β1-integrin cooperates with VEGFR3 to sense perfusion-induced stretching and to induce angiocrine HGF, IL-6, TNF, and MMP9 (Lorenz et al. 2018). Since zone 2 is the main proliferative region during injury, how these mechanical cues are sensed across different zones of the lobule and translated into spatially coordinated proliferation remained unclear.

Zhang and colleagues now uncover a mechanosensory axis that redefines our understanding of liver regeneration (Zhang et al. 2026). Through integration of single-cell transcriptomics, lineage tracing, and genetic perturbations, they identify DPP4-positive zone 2 hepatocytes as a mechanosensitive subpopulation that drives liver regrowth. Using *Dpp4*-*CreER*^*T2*^ lineage tracing, they demonstrate that DPP4-positive cells are highly proliferative and give rise to large clones that spread from zone 2 toward periportal and pericentral regions after partial hepatectomy. Biophysical mapping with atomic force microscopy and fluorescence lifetime imaging reveals that zone 2 hepatocytes undergo the most pronounced hypertrophy, accompanied by increased membrane tension, cortical contractility, and stiffness. These cells express high levels of PIEZO1, which mediates calcium influx and cell cycle activation in response to mechanical strain. Thus, DPP4-positive hepatocytes are not merely passive responders to injury but are intrinsically primed to detect and translate localized mechanical cues into a proliferative advantage.

**Fig. 1:**
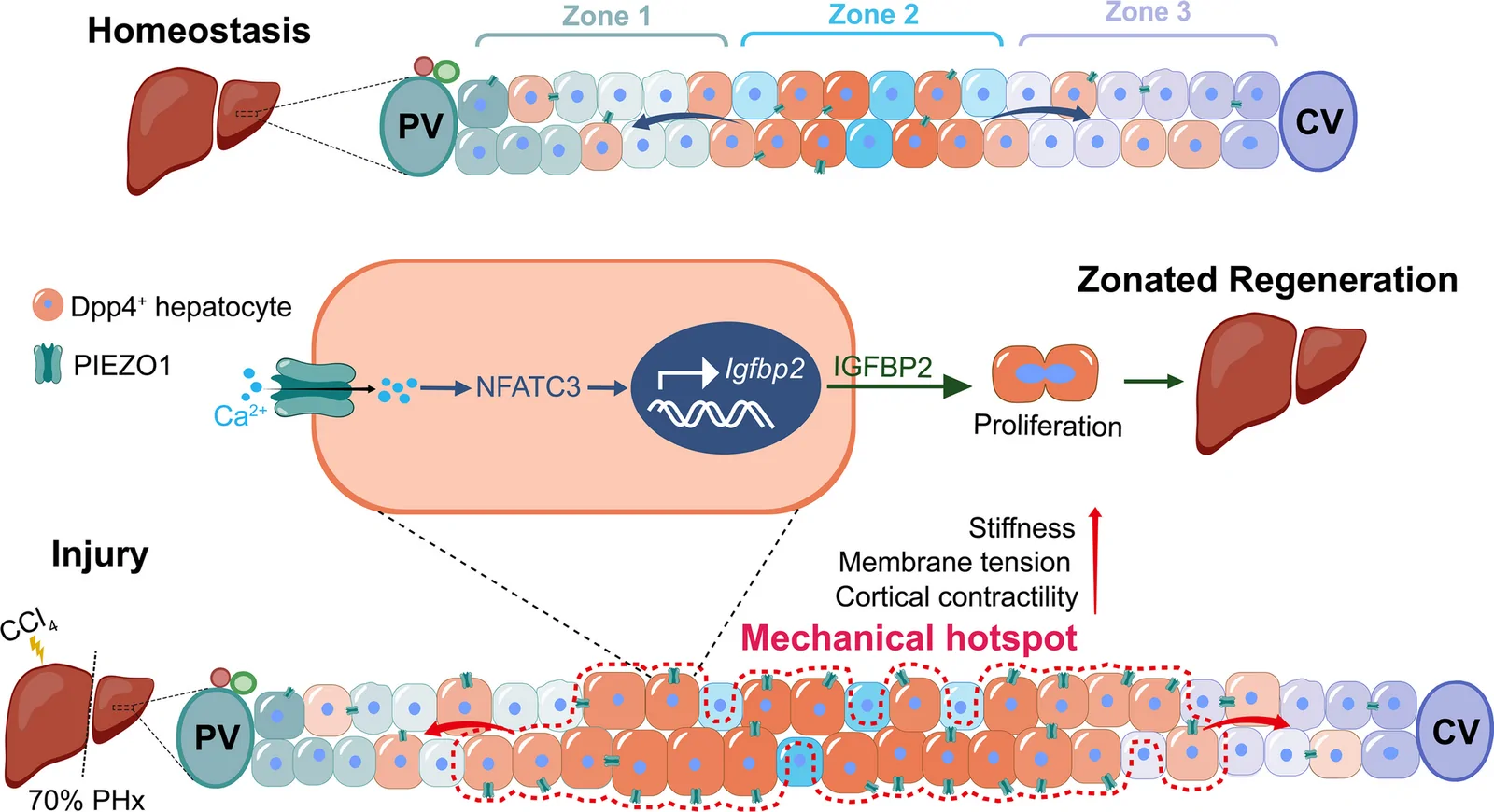
Liver regeneration governed by zonated PIEZO1 mechanotransduction. Study by Zhang et al. (2026) demonstrates that DPP4⁺ zone 2 hepatocytes act as a mechanosensory population in the liver. Under homeostatic conditions, hepatocytes are organized into zone 1, zone 2, and zone 3 along the porto-central axis. After injury such as 70% partial hepatectomy (PHx) or CCl₄ treatment, increased tissue stiffness, membrane tension, and cortical contractility create a mechanical hotspot in the mid-lobular region. DPP4⁺ zone 2 hepatocytes sense this stress through PIEZO1, which mediates Ca^2^⁺ influx and activates NFATC3-dependent expression of *Igfbp2*. Secreted IGFBP2 promotes hepatocyte proliferation and clonal expansion, leading to zonated regeneration and restoration of liver mass. Genetic deletion and gain-of-function show that the PIEZO1-NFATC3-IGFBP2 axis is required and sufficient for zone 2-mediated liver regrowth, as depicted in the figure (Zhang et al. 2026)

Building on these biophysical and molecular observations, the authors next interrogate the functional necessity of PIEZO1 through targeted genetic manipulations. Using hepatocyte-specific and zone 2-restricted genetic deletions, the authors demonstrate that PIEZO1 is indispensable for regeneration. Depleting *Piezo1* in DPP4-positive cells suppresses hepatocyte proliferation, delays liver-mass restoration, and impairs recovery after both partial hepatectomy and centrilobular toxin injury, whereas loss of *PIEZO1* in liver sinusoidal endothelial cells has no effect. Mechanistically, PIEZO1 drives proliferation through NFATC3-dependent induction of insulin-like growth factor binding protein 2 (IGFBP2). Overexpression of *Igfbp2* rescues the regenerative defect in *Piezo1*-deficient mice. Conversely, zone 2 restricted *PIEZO1* gain-of-function enhances *Igfbp2* expression, accelerates clonal expansion, and improves liver regrowth. These findings establish a link between tissue mechanics and the spatial coordination of liver regeneration, identifying PIEZO1 as a mechanotransducer that converts localized physical stress into a proliferative program. The PIEZO1-NFATC3-IGFBP2 axis provides a zone 2-specific upstream mechanism that links injury-induced mechanical forces to activation of a proliferative program. Although the mechanisms that initiate liver regeneration remain insufficiently established, the evidence that IGFBP2 overexpression fully rescues the regenerative defect places this axis at a functionally critical node for zone 2 hepatocyte proliferation (Fig. 1).

This elegant study provides a compelling paradigm for how mechanical forces guide liver regeneration, yet several key questions remain. First, whether PIEZO1 contributes to steady-state zonation is unclear, as deficiency might alter markers such as GS, E-CAD, or DPP4. Although hypertrophy, matrix remodeling, and hemodynamic forces are all plausible contributors to the mechanical environment of zone 2, the precise cue that directly activates PIEZO1 remains unidentified. It is still unclear which input predominates or whether they converge downstream. Furthermore, YAP and TAZ activity is partially reduced in *Piezo1* KO livers; however, whether PIEZO1-mediated calcium influx directly modulates YAP/TAZ phosphorylation and nuclear translocation or acts through additional intermediates during liver injury remains unclear. Moreover, as DPP4-positive cells expand and generate progeny that settle in zones 1 and 3, it remains unclear whether these newly positioned cells retain the same zone 2 identity or adopt the characteristics of their new microenvironment. Additionally, why PIEZO1 selectively marks DPP4-positive cells rather than being uniformly expressed across zone 2 warrants exploration. PIEZO1 is also expressed at lower levels in zone 3 hepatocytes; whether it serves a distinct role in zone 3 physiology or injury response merits investigation. Finally, comparing PIEZO1 function across tissues will determine whether mechanotransduction follows a conserved logic or is tailored to tissue-specific contexts.

## Conclusions

This study establishes mechanical force sensing as a central driver of liver regeneration, identifying PIEZO1 as a molecular switch that converts physical strain into a proliferative program. Importantly, this mechanosensory function is spatially restricted, as zone 2 hepatocytes are uniquely equipped to perceive and respond to mechanical cues, providing a framework for the spatial logic of liver repair. In this context, mechanotransduction is positioned alongside chemical signaling as a complementary control mechanism. Therapeutically, the PIEZO1-NFATC3-IGFBP2 axis offers a promising target for acute liver failure and chronic liver disease, given that enhancing this pathway accelerates recovery. However, its efficacy and safety in chronic or fibrotic liver disease remain to be evaluated in additional disease settings. Small-molecule agonists such as Yoda1 can activate PIEZO1, but they exhibit potential off-target effects and poor pharmacokinetic profiles. Ultimately, this research redefines the liver as a mechanosensitive organ in which physical forces are integral to restoring form and function.

## Data Availability

Not applicable.
